# Microbial Interrelationships across Sites of Breastfeeding Mothers and Infants at 6 Weeks Postpartum

**DOI:** 10.3390/microorganisms10061155

**Published:** 2022-06-02

**Authors:** Erin C. Davis, Mei Wang, Sharon M. Donovan

**Affiliations:** 1 Division of Nutritional Sciences, University of Illinois, Urbana, IL 61801, USA; erin_davis1@urmc.rochester.edu; 2 Department of Food Science and Human Nutrition, University of Illinois, Urbana, IL 61801, USA; meiwang@illinois.edu

**Keywords:** human milk, infant microbiome, human milk microbiome, breastfeeding

## Abstract

Infancy is a critical life stage for the establishment of the gut microbiome. Human milk contains a unique microbial ecosystem that serves as a continuous source of commensal bacteria for the infant. However, the origin of the human milk microbiota, how it is influenced by breastfeeding exclusivity, and its role in infant gut microbiota assembly are not clear. To interrogate these questions, we examined the relationships among fecal, oral, breast skin, and human milk microbiota of 33 exclusively breastfeeding (EBF) and mixed-feeding (MF; human milk + infant formula) mother–infant pairs at 6 weeks postpartum. Here, we show that MF infants have a significantly more diverse oral microbiome comprised of lower relative abundances of *Streptococcus* and *Gemella* and higher abundances of *Veillonella*. Using both SourceTracker2 and FEAST, we demonstrate breast skin and infant saliva as the principal contributing sources to the human milk microbiota. Of the sampled sites, human milk and maternal stool were predicted to contribute the largest fraction to the infant fecal microbiome, but the majority of the community was estimated to arise from unknown sources. Lastly, we identified twenty-one significant co-occurrence relationships between bacteria in human milk and on other maternal and infant body sites. These results demonstrate several unique microbial interrelationships between breastfeeding dyads, providing insight into potential mechanisms of microbial assembly in early life.

## 1. Introduction

Microbial colonization during infancy plays an essential role in immune maturation, development of the gastrointestinal tract, and metabolic programming [1,2]. Aberrations in colonization or early dysbiosis have been associated with a number of non-communicable diseases in childhood, including obesity [3], inflammatory bowel disease [4], food allergy [5], and asthma [6], highlighting the long-term impact of early microbial colonization. Among other perinatal factors, infant diet plays a principal role in shaping microbial composition [1,7,8,9,10]. Human milk (HM) serves as a critical link between mother and infant, delivering bioactive components uniquely suited to drive microbial development [7,8]. HM contains a diverse microbial ecosystem, which serves as a continuous source of bacteria for the recipient infant [11,12,13,14,15,16,17]. While knowledge of the composition of the microbial community in HM has greatly increased in recent years, its origin, relationship to other microbial sites, and role in the development of the infant gut microbiota remain unclear [18,19].

The composition of the HM microbiota is unique among mothers and is influenced by delivery mode [14], geography [15,20], maternal health status [14,16], and breast pump usage [17,21]. This variation within the HM microbiota may be responsible, in part, for inter-individual variation within the gut microbiota of breastfed infants [12,13,22,23]. The HM microbiome is hypothesized to originate from maternal and infant body sites, including the maternal and infant oral cavity, breast skin, and the maternal gastrointestinal tract [18,19,24]. Evidence from rodent studies suggests that bacteria are transported via dendritic cells from the maternal gut to the mammary gland during pregnancy and lactation via an enteromammary pathway [25,26,27]. Vertical transmission, mainly of bifidobacterial species and strains, from the maternal to infant gut during lactation has also been documented and further supports the concept of an endogenous origin for a portion of the HM microbiota [23,28,29]. Multiple studies have also shown strong associations between various combinations of these sites [30,31,32,33,34,35], yet few studies have evaluated all hypothesized microbial sources in the same mother–infant pairs.

Infant oral, and likely skin, bacteria are thought to contribute to the HM microbiota via retrograde backflow of milk into the mammary ducts during suckling [36]. Previous findings demonstrate that oral microbiota composition differs between exclusively breastfeeding (EBF) and formula-feeding infants [37,38]. Therefore, it stands to reason that the oral microbiota of EBF and mixed-feeding (MF) infants, those consuming both HM and infant formula, may also differ. If so, differences in the infant oral microbiota may subsequently influence the microbial composition of their mothers’ milk, thus inducing more variation in this community among women. However, the impact of breastfeeding exclusivity on the composition of the HM microbiota has only been investigated in a couple of studies [32,39], neither of which explored the infant oral microbiota.

Further insight into the origin of the HM microbiota, factors that influence its composition, and its contribution to infant microbial assembly is necessary to advance our understanding of how the gut microbiome is shaped in early life. Herein, interrelationships among fecal, oral, breast skin, and HM microbiota of breastfeeding mothers and infants, as well as evaluating how these communities are influenced by feeding modes, are investigated.

## 2. Materials and Methods

### 2.1. Subjects

Healthy mother–infant pairs (N = 33 pairs; *n* = 20 EBF, *n* = 13 MF) were recruited and enrolled in the Microbial Interrelationships between Mothers and Infants by Mode of Feeding (MIMI) Study between April 2016 and February 2018. The MIMI Study was a small subproject of the STRONG Kids 2 Cohort Study [40], a large birth cohort study aimed at assessing predictors of dietary habits and weight trajectories in early childhood. Additionally, in order to increase the number of MF infants, we also recruited a small number of mother–infant pairs from the general community of Champaign-Urbana, IL. All STRONG Kids 2 participants were offered participation in the MIMI Study until we reached our target sample size for each feeding mode. Dyads were screened for eligibility, specific to the MIMI Study, and enrolled between 5 and 6 weeks (wks) postpartum. To enroll, infants had to have been EBF since birth or actively MF (consuming both human milk + infant formula) during the 7 days prior to study enrollment. Exclusion criteria included pre-term or multiple births, infant chronic medical conditions that affected feeding (i.e., down syndrome, congenital heart disease, cleft palate, cystic fibrosis, hormone deficiency), mastitis at screening, maternal or infant probiotic usage since delivery, antibiotic usage in the 7 days prior to sample collection, inflammatory bowel disease, irritable bowel syndrome, and oral or gastrointestinal infection at screening. Study procedures were approved by the University of Illinois at Urbana-Champaign Institutional Review Board (#16525), and written informed consent was obtained by all mothers.

### 2.2. Study Design and Data Collection

The study was cross-sectional and consisted of 2 visits between 5 and 7 weeks postpartum, the first of which consisted of study enrollment procedures. Between visits, mothers collected biological samples and completed a study questionnaire containing information about demographics, medical history, physical health, and infant feeding practices. The research staff returned for a second visit to retrieve samples and collect height/length and weight measurements for mother and infant. STRONG Kids 2 also had a study visit at 6 weeks postpartum, at which time maternal and infant stool samples and height and weight measurements were collected; therefore, these were also utilized for the MIMI study to avoid duplicate collection. These samples and data were collected in the same way as those participants only enrolled in the MIMI Study. All samples collected under either the STRONG Kids 2 Study or the MIMI Study were handled using the same procedures and processed and analyzed together.

### 2.3. Sample Collection

Samples were collected at home by mothers at 6 wks postpartum using a detailed protocol provided by research staff. Mothers were instructed to collect all samples within a 24-hour (h) period. Upon collection, each sample was immediately placed in the mother’s home freezer, and all samples were transported to the laboratory on ice and stored at −80 °C until analysis.

Stool samples. Freshly voided maternal stool samples were collected using a commode specimen system (ThermoFisher Scientific, Waltham, MA, USA), and infant stool samples were retrieved from one diaper. Samples were transferred to sterile 50 mL conical tubes using a disposable sterile spoon.

Saliva. Infant saliva was collected when infants had not consumed human milk or formula for 1.5–2 h. Two separate CS-1 sponges (DNA Genotek Inc., Kanata, ON, Canada) were placed under the infant’s tongue to soak up pooled saliva for 30 s (sec), and the head of the swabs was clipped into an OMNIgene•DISCOVER (OM-501) kit (DNA Genotek Inc.). Maternal saliva was collected in the morning before eating, drinking, or teeth-brushing by spitting into an OM-501 kit.

Breast skin. Samples were collected when mothers had not showered for 6–8 h or expressed milk (breast pump or infant feeding at breast) for 2 h. Mothers dampened a nylon FLOQswab (Copan Diagnostics, Murrieta, CA, USA) in sterile phosphate-buffered saline (PBS) and rolled the swab over the nipple, areola, and a 1-inch radius of breast skin past the areola for 30 s. The head of the swab was then cut into a sterile tube containing 2 mL of PBS. Breast skin samples were always collected prior to HM collection discussed below.

Human milk. Samples were collected when mothers had not expressed milk for 2 h. To decrease the possibility of contamination during collection, mothers put on a pair of individually packaged, sterile gloves (Henry Schein, Melville, NY, USA) and cleaned their breast with a povidone-iodine swab followed by an alcohol swab. The first pair of sterile gloves was replaced, and after discarding roughly the first teaspoon of milk, mothers manually expressed 15 mL of breast milk into a sterile cup or tube [14,41].

### 2.4. DNA Extraction

DNA was extracted from all sample types using a QIAamp Fast DNA Stool Mini Kit (Qiagen no. 51604, Valencia, CA, USA) in combination with bead-beating and the FastPrep-24 System (MP Biomedicals, Carlsbad, CA, USA). For both maternal and infant stool samples, the extraction was performed using 200 mg of stool, as previously described [42]. For maternal saliva, the OM-501 tubes containing free saliva or infant swabs and stabilization reagent were incubated at 50 °C for 1 h per the manufacturer’s instructions. Infant saliva was retrieved from swabs, and DNA was extracted from 500 µL of maternal or infant saliva using the same methods as the fecal samples. DNA from saliva was eluted in 50 µL of ATE buffer after a 5-minute (min) incubation period.

For skin samples, the head of the swab was cut directly into a Lysing Matrix E bead-beating tube (MP Biomedicals, Solon, OH, USA), and the remainder of free liquid from the sample collection tube was divided among 4 remaining bead-beating tubes (500 µL per tube). Next, 1 mL of Inhibit EX buffer was added to the tube with a swab, and 900 µL was added to tubes containing free liquid. All tubes were shaken for 60 s at 6 m/s on a FastPrep-24 System (MP Biomedicals). The remaining steps of extraction followed those for saliva and stool samples; however, the contents of all bead-beating tubes for one participant were passed through the same spin column to pool DNA. DNA was eluted in 35 µL ATE buffer after a 5 min incubation.

Human milk samples were thawed on ice, and 3 mL of milk were centrifuged at 14,000× *g* for 15 min at 4 °C. The fat layer was removed, whey discarded, and the cell pellet resuspended in 500 µL of TE buffer (10 mM Tris, 1 mM EDTA, pH 8.0). DNA was extracted using methods similar to those previously described [43]. Briefly, 100 µL of a lytic enzyme mixture containing 50 µL lysozyme (10 g/L in nuclease-free water), 12 µL mutanolysin (12.5 KU/mL in nuclease-free water), 3 µL lysostaphin (4000 U/mL in 20 mM sodium acetate), and 35 µL TE buffer was added to samples and placed on a dry heat block at 37 °C for 1 h. The lysate was further subjected to mechanical disruption by bead-beating in a Lysing Matrix E tube with glass beads (MP Biomedicals) for 1 min at 5 m/s on a FastPrep-24 System (MP Biomedicals). The remainder of the extraction followed the manufacturer’s instructions for the QIAamp Fast DNA Stool Mini Kit with one extra step—100 μL of 3M sodium acetate (pH 5.5) prior to the addition of ethanol to help DNA bind to the column. DNA was eluted in 35 µL of AE buffer after a 5 min incubation.

For all sample types, DNA concentration was quantified using a Qubit dsDNA Assay Kit (Life Technologies, Carlsbad, CA, USA). DNA was stored at −20 °C until PCR amplification and sequencing.

### 2.5. PCR Amplification and 16S rRNA Sequencing

Extracted DNA from fecal, oral, skin, and human milk samples were submitted to the Roy J. Carver Biotechnology Center at the University of Illinois at Urbana-Champaign for PCR amplification and sequencing, as previously described [44]. Briefly, the V3-V4 region of bacterial 16S rRNA genes was amplified using primers V3f (5′-CCTACGGGNGGCWGCAG-3′) and V4r (5′-GACTACHVGGGTATCTAATCC-3′) [45] on a Fluidigm Biomark HD platform (Fluidigm Corporation, South San Francisco, CA, USA), with temperature profiles as previously described by Muturi et al. [46]. Amplicons from 48 separate reactions were pooled for each sample, and DNA concentration was measured using a Qubit 3.0 fluorometer (Thermo Fisher Scientific, Waltham, MA, USA). Amplicon size was confirmed by running the samples on a fragment analyzer (Advanced Analytical Technologies, Ankeny, IA, USA). The amplicons were mixed in equimolar concentrations and run on a 2% agarose E-gel (Thermo Fisher Scientific), and the band of expected size (c.460 bp) was excised from the gel. DNA was extracted with a Qiagen Gel Extraction Kit, and extracted DNA was run on an Agilent 2100 bioanalyzer (Agilent Technologies, Santa Clara, CA, USA) to confirm an appropriate profile and determine the average size. Sequencing was then performed using an Illumina MiSeq flow cell for 251 cycles from each end of the fragments with MiSeq Reagent version 2 (2 × 250 nt paired-end reads; Illumina, San Diego, CA, USA) [47]. Sterile DNA-free water from our laboratory and the Roy J. Carver Biotechnology Center were included as both dilution and sequencing controls.

### 2.6. Sequence Processing

Paired-end sequence reads were processed using QIIME 2 version 2018.11 [48]. Raw sequences were demultiplexed, trimmed, and quality-filtered using the q2-demux plugin and denoised using DADA2 [49]. Amplicon sequence variants (ASVs) were aligned with mafft [50], and the phylogenetic tree was constructed using fasttree [51]. Amplicon sequence variants (ASVs) present in less than 2 samples and comprised of less than 20 total reads across all samples were removed. Any reads that were ‘unassigned’ after initial preliminary taxonomic analyses were removed, and sequences were rarefied to 7000 reads per sample prior to core diversity analyses. Alpha diversity (Shannon index, observed features, and Faith phylogenetic diversity) and beta diversity distance metrics (Bray–Curtis dissimilarity, unweighted and weighted UniFrac) were calculated from the filtered and rarefied ASV table to standardize sampling effort. Taxonomy was assigned to ASVs using the q2-feature-classifier [52] against the SILVA 132 reference database [53]. Reads aligning to Archaea or chloroplast were removed prior to the calculation of relative abundance within QIIME2.

### 2.7. Statistical Analyses

All analyses were conducted using SAS 9.4 (SAS Institute Inc., Cary, NC, USA) unless otherwise stated. Differences in demographic characteristics between EBF and MF groups were analyzed using either a Kruskal–Wallis test or Fisher’s exact test. Differences in microbial structure between body sites and between feeding groups (within each body site) were evaluated by permutational multivariate analysis of variance (PERMANOVA) performed on Bray–Curtis Dissimilarity and weighted and unweighted UniFrac distances within QIIME2. Differences in alpha diversity (Shannon index) between body sites were assessed using a Kruskal–Wallis test within QIIME2. For each body site, differences in alpha diversity indices (Shannon index, Faith phylogenetic diversity, observed features) between feeding groups were analyzed using a one-way analysis of variance (ANOVA) via MIXED (normally distributed or transformed data) or GLIMMIX (non-normally distributed data) procedures in SAS. A Poisson distribution was utilized when models were run with the GLIMMIX procedure. Differences in the relative abundance of bacterial genera between feeding groups within each microbial site were assessed using the GLIMMIX procedure utilizing a beta distribution appropriate for proportional data. Statistical tests of differential abundance were only carried out on taxa present in at least 20% of samples and at a mean relative abundance of ≥0.1% across all samples within a microbial site. All zero values were replaced with a pseudocount of 1 × 10^−6^ prior to analysis. Delivery mode prevalence and maternal BMI differed between EBF and MF groups. Therefore, when analyzing alpha diversity or differential abundance between feeding groups in HM, infant saliva, breast skin, and infant stool, delivery mode was included as a covariate. For the same analyses in maternal stool and saliva, maternal BMI at the time of sample collection (6 wks postpartum) was included as a covariate. Level of significance was set at *p* ≤ 0.05 and trends accepted at 0.05 < *p* < 0.10.

### 2.8. Microbial Source Prediction Analysis

SourceTracker2 was used to estimate the proportions of bacteria from sampled sites to both the HM and infant fecal microbiota [54]. The first analysis investigated the fraction of ASVs in HM (sink sample) that was contributed by ASVs in maternal stool and saliva, infant saliva, and breast skin (source environments). Next, we performed a similar analysis with infant stool as the sink community, estimating the fraction of ASVs contributed by communities in HM, maternal stool and saliva, infant saliva, and breast skin. EBF and MF source samples were collapsed into one group for each body site; thus, we did not run SourceTracker2 on samples from each feeding group separately. A filtered, rarefied ASV table was used for analysis. No further rarefaction was specified during analysis. In addition to SourceTracker2, we also utilized FEAST (fast expectation-maximization for microbial source tracking) [55] as another method to predict microbial source contributions to both HM and infant stool microbiota, using the same source–sink combinations as previously stated. We first ran FEAST by assigning a distinct set of source samples to each sink sample, i.e., the fraction of contribution was only estimated from source environments within a given dyad. This will be referred to as ‘FEAST—Individual’. Next, we estimated the fraction of ASVs contributed by the same source environments for all sink samples, i.e., all source samples from every dyad were used to predict source site contribution to one individual HM or infant stool sample. This will be referred to as ‘FEAST—Group’. The second FEAST method was utilized as it was more similar to the SourceTracker2 results. Additionally, as each microbial site profiled in this study is fairly distinct, FEAST—Group may also be useful in providing a broad insight into the origin site(s) for ASVs in HM and infant stool.

### 2.9. Co-Occurrence Network Analysis

CoNet, a Cytoscape 3.0 application [56], was used to assess bacterial co-occurrence and co-exclusions relationships between HM and each subsequent microbial site. Networks were only evaluated at the genus level using relative abundance. An ensemble approach, combining Pearson correlation, Spearman correlation, Mutual Information, Bray–Curtis dissimilarity, and Kullback–Leibler dissimilarity, was utilized for network inference [57]. Network construction included a bootstrap-renormalization step to account for compositionality in the data. Once calculated, *p*-values were merged using Brown’s method, and Benjamini–Hochberg multiple testing correction was employed to control the false discovery rate. Five separate networks between HM and each additional site were created and merged after analysis for visualization.

## 3. Results

### 3.1. Subject Characteristics

The demographic characteristics of the study subjects are listed in Table 1. On average, mothers were 32 years old and had a BMI of 22.9 kg/m^2^ prior to pregnancy and 24.8 kg/m^2^ at the time of sample collection. Pre-pregnancy BMI was significantly higher among MF compared to EBF mothers but did not differ between groups at 6 wks postpartum. Most mothers delivered infants vaginally (73%); however, mothers in the MF group were more likely to deliver via cesarean section (C-section). Infants who were MF received HM for, on average, 67% of feedings. About half of the mothers (48%) responded that they regularly pumped breast milk, with no difference between feeding groups.

### 3.2. Bacterial Composition among Maternal and Infant Body Sites

**Figure 1:**
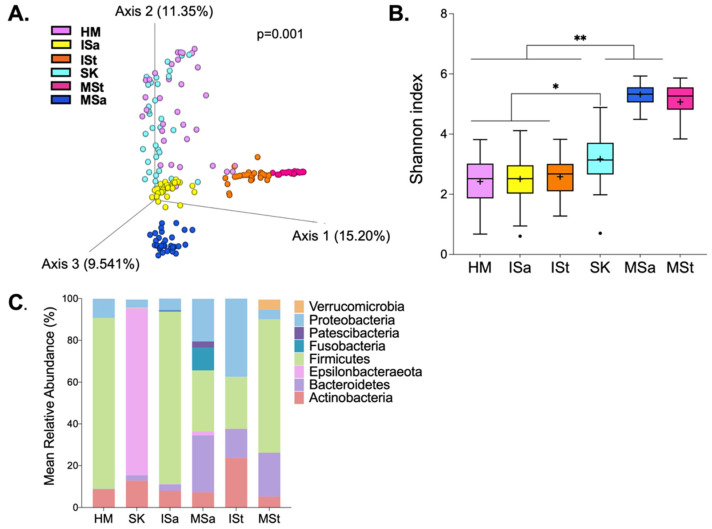
Microbial composition between maternal and infant body sites. (**A**) Beta diversity. Differences in overall microbial structure among sites were analyzed using PERMANOVA performed on Bray–Curtis dissimilarity distances. (**B**) Alpha diversity. Differences in Shannon diversity were analyzed using a Kruskal–Wallis test and are significantly different at ** *p* ≤ 0.001 and * *p* ≤ 0.01. (**C**) Bacterial phyla present in at least 20% of samples and at ≥0.1% mean relative abundance across samples within each body site. HM, human milk; SK, breast skin; ISa, infant saliva; MSa, maternal saliva; ISt, infant stool; MSt, maternal stool.

Overall, DNA from 198 samples were sequenced, generating 8,250,778 sequence reads after quality filtering. One infant stool sample was removed from further analysis due to low read counts. Among the remaining samples, the mean ± SEM read count was 41,881 ± 2194 reads per sample. We first explored differences in microbial structure and composition among HM, breast skin (SK), infant stool (ISt), infant saliva (ISa), maternal saliva (MSa), and maternal stool (MSt). As expected, the overall microbial structure differed among all communities (Figure 1A). Maternal and infant stool samples clustered closely together, both within and between communities. Notably, HM clustered most closely to SK. There appeared to be greater inter-individual variation in HM, SK, and ISa compared to stool samples and MSa. As we anticipated, Shannon diversity also differed significantly among sites (Figure 1B). MSa and MSt communities were significantly more diverse than those of HM, SK, ISa, and ISt (*p* < 0.001). Diversity within ISa and ISt was similar to that of HM samples, but all three communities were significantly less diverse than SK (*p* < 0.01). Eight phyla, present at a mean relative abundance ≥0.1%, were detected across the six microbial communities (Figure 1C). Firmicutes dominated HM, SK, and MSt communities. MSa harbored the greatest number of bacterial phyla, including the largest proportion of Fusobacteria. Though detected in some samples of other sites, Verrucomicrobia and Epsilonbacteraeota were only present in more than 20% of samples within MSt and MSa, respectively.

### 3.3. Bacterial Diversity between Feeding Groups

**Figure 2:**
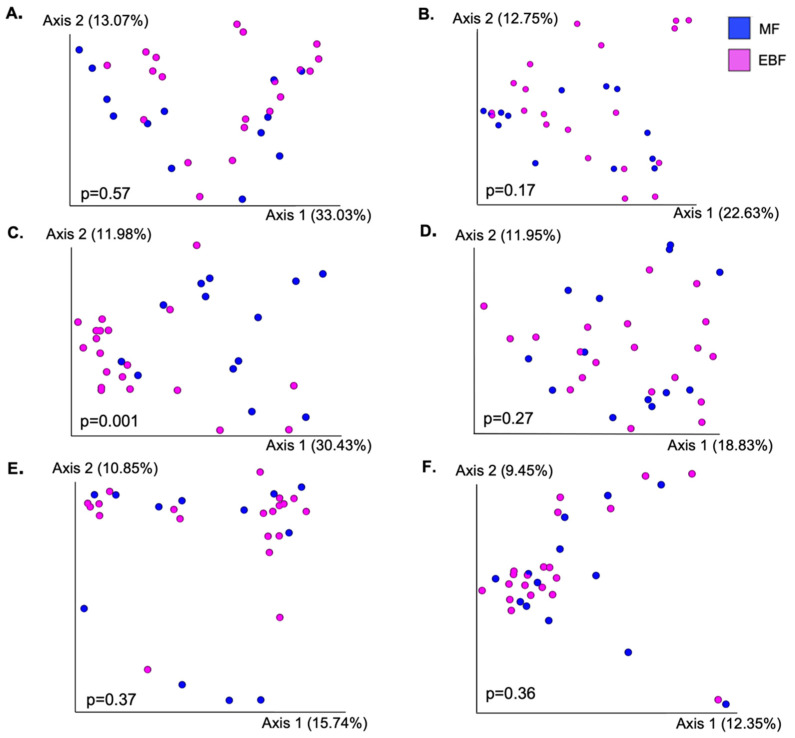
Microbial structure between feeding groups on different maternal and infant body sites. Differences in overall community structure between feeding groups were analyzed using PERMANOVA performed on Bray–Curtis dissimilarity distances. (**A**) Breast skin; (**B**) human milk; (**C**) infant saliva; (**D**) maternal saliva; (**E**) infant stool; (**F**) maternal stool.

Next, we compared community structure (Figure 2) and alpha diversity (Table 2) between EBF and MF mothers or infants within each body site. The only difference in overall bacterial composition, as measured by Bray–Curtis dissimilarity, between EBF and MF groups was in ISa (*p* = 0.001). We did identify additional trends and significant differences when assessing differences in UniFrac distances (Table S1). This was most notable in HM; however, the significant difference in microbial structure between EBF and MF was largely driven by one HM sample; when this sample was removed, the results were no longer significant. Results marginally differed when comparing beta diversity using weighted and unweighted UniFrac (Table S1). The oral microbiota of MF infants was significantly more diverse than that of EBF infants; MF infants had both a higher Shannon index as well as a greater number of observed features (*p* < 0.05). Shannon diversity was also greater in the SK of MF mothers (*p* < 0.05). There were no differences in the alpha diversity of HM, MSa, MSt, or ISt communities between feeding groups.

### 3.4. Taxonomic Composition between Feeding Groups

Next, we assessed differences in the relative abundance of the most abundant bacterial genera between feeding groups within each body site (Table 3 and Tables S2–S7). At the genus level, HM was dominated by *Staphylococcus* and *Streptococcus*, which together accounted for an average of about 77% of the bacterial community. Composition varied greatly among mothers, with 78 different genera being identified within HM samples, only 10 of which were present at mean relative abundance greater than 0.1% and detected in more than 20% of samples. The remainder of the community consisted of genera such as *Corynebacterium*, *Rothia*, *Gemella*, *Veillonella*, and *Cutibacterium*, all of which have been consistently identified as members of the HM microbiota [13,14,15,16,17]. *Streptococcus* tended to be higher in the HM of EBF mothers (*p* = 0.10) compared with MF mothers; however, the abundance of no other genera differed in HM between the two feeding groups.

Similar to HM, the bacterial community on SK was also largely dominated by *Staphylococcus* and *Streptococcus* as well as *Corynebacterium 1*, comparable to previous findings [33]. Notably, the six most abundant genera identified in SK samples were also among the most abundant in HM samples. *Anaerococcus* tended to be higher in the SK of MF mothers (Table S3), while *Streptococcus* tended to be higher in the SK of EBF mothers (*p* = 0.07); however, this trend for *Streptococcus* was no longer apparent after controlling for delivery mode. Though the majority of ISa was comprised of only a small number of bacterial genera, a few differences were identified between EBF and MF infants. The saliva of EBF infants harbored higher relative abundances of both *Streptococcus* and *Gemella*, while *Veillonella* was more abundant among MF infants.

The most abundant genera present in ISt were *Bifidobacterium*, *Escherichia-Shigella*, and *Bacteroides*. Surprisingly, feeding mode had no effect on the abundance of any genera in ISt. Lastly, a number of trends and significant differences were identified between EBF and MF mothers in the relative abundance of the most abundant genera present in MSa and MSt (Table 3, Tables S6 and S7); however, these differences were not reflected across any other body sites.

### 3.5. Microbial Sources Contributing to Human Milk and Infant Fecal Microbiota

**Figure 3:**
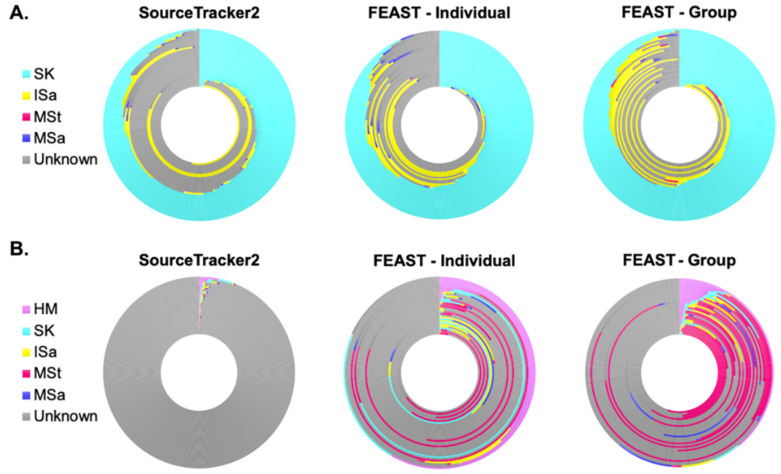
Proportions of human milk and infant stool bacterial communities estimated to originate from maternal and infant body sites. Each ring represents an (**A**) individual human milk (*n* = 33) or (**B**) infant stool (*n* = 32) sample. Colors correspond to the relative amount (%) of amplicon sequence variants (ASVs) predicted to come from the body site of the corresponding color. Contributions were estimated using SourceTracker2 and two methods of FEAST. FEAST—Individual results were estimated using different groups of source samples for each sink (human milk or infant stool); contributions were only estimated from samples within the same dyad. FEAST—Group results were estimated using the same group of source samples for each sink sample (human milk or infant stool); contributions were estimated from all samples within a given body site. HM, human milk; SK, breast skin; ISa, infant saliva; MSa, maternal saliva; MSt, maternal stool.

Though we saw no differences in the bacterial composition of HM and ISt based on feeding mode, we still sought to assess the relative contributions of each sampled site to both of these communities. Using SourceTracker2 [54], we were able to predict sites contributing to more than half of the ASVs present in the HM microbiota, with notable variation among samples (Figure 3A, Table S8). On average, 50% of the ASVs in HM were estimated to come from SK, with the next highest fraction from a sampled sight coming from ISa. However, roughly 41% of ASVs in HM were also predicted to come from unknown sources, i.e., sites not sampled or considered in this analysis. MSa and MSt were both predicted to contribute less than 1%, on average, of the ASVs in HM. We also utilized FEAST [55], another microbial source tracking framework, to identify source environments and their estimated contributions. Two different models were run with different sets of source samples. FEAST—Individual estimated the fraction of contribution from source samples to a sink sample within an individual dyad, so each sink sample had a distinct set of potential sources. FEAST—Group, on the other hand, estimated the fraction of ASVs contributed by the same set of source samples for all sink samples—all source samples from every study participant served as potential sources. For example, all MSt samples were used as source samples to predict the fraction of ASVs in one individual HM sample coming from MSt. Both FEAST—Individual and FEAST—Group were more efficient at predicting microbial site contribution compared to SourceTracker2 (Figure 3A, Table S8), decreasing the fraction of ASVs coming from unknown sources by an average of 10% and 17%, respectively. Still, the patterns for FEAST and SourceTracker2 were similar when HM was the sink sample, with SK estimated to contribute the largest fraction of bacteria, followed by unknown sources and ISa. Maternal stool and saliva were still predicted to contribute only a small amount of ASVs to HM.

Conversely to what we identified in HM, SourceTracker2 predicted the sampled maternal and infant microbial sites to contribute just over 1% of the ASVs found in ISt (Figure 3B, Table S8). Though all sites were estimated to contribute, HM was estimated to contribute the greatest proportion of ASVs to the bacterial community in ISt. FEAST was able to predict microbial sources for a much higher fraction of ASVs in ISt. Estimated contributions increased from all microbial sources, though a notably higher predicted fraction came from MSt. Even with these improvements, FEAST results identified that more than 60% of the ASVs in infant stool, on average, arose from unknown sources.

### 3.6. Bacterial Co-Occurrence Patterns between Human Milk and Maternal and Infant Body Sites

**Figure 4:**
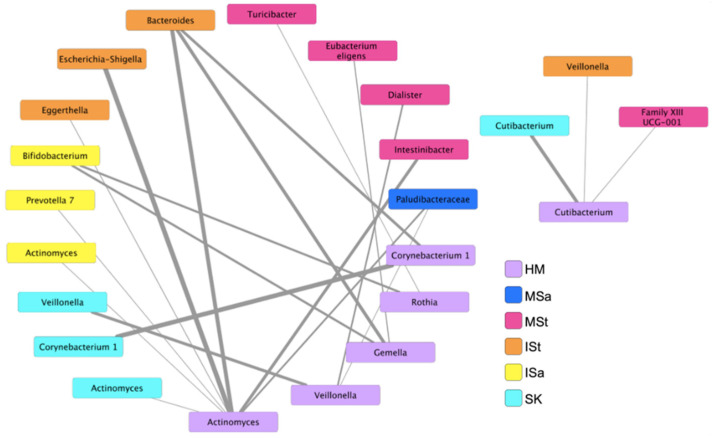
Co-occurrence patterns between bacterial genera in human milk and maternal and infant body sites. Only significant associations are shown (*p* ≤ 0.05). Widths of lines represent significance of association, with lower *p*-values corresponding to thicker lines. HM, human milk; SK, breast skin; ISa, infant saliva; MSa, maternal saliva; ISt, infant stool; MSt, maternal stool.

To further explore microbial interactions between mothers and infants, we utilized an ensemble approach in CoNet to investigate bacterial networks, at the genus level, between HM and all other maternal and infant body sites. Twenty-one significant co-occurrence patterns were identified between bacteria in HM and those on other sites (Figure 4). No co-exclusion patterns were detected. Six nodes emerged in HM, including *Corynebacterium 1*, *Cutibacterium*, *Gemella*, *Rothia*, *Veillonella*, and *Actinomyces*. While both SourceTracker2 and FEAST estimated that the majority of the ISt microbial community originated from unknown sources, a number of associations between bacterial genera in HM and ISt were identified in this analysis. *Actinomyces* in HM were associated with infant fecal *Escherichia-Shigella*, *Eggerthella*, and *Bacteroides*, and *Gemella* and *Corynebacterium* in HM were also associated with *Bacteroides* in ISt. Interestingly, relationships between *Bifidobacterium* in ISa and *Gemella* and *Rothia* in HM also emerged in the network. Unlike other sites, co-occurrence patterns between bacteria in HM and SK were only discovered between the same genera (*Cutibacterium*, *Veillonella*, *Actinomyces*, and *Corynebacterium*), supporting the notion of SK as a principal contributor to the HM microbiota. Additional relationships were also identified between genera in HM and various members of the Firmicutes phylum in MSt.

## 4. Discussion

Assembly of the early life gut microbiota is a complex process that is intricately connected to long-term immunologic and metabolic programming [1]. Human milk contains a diverse microbial ecosystem thought to shape the gut microbiota composition of breastfed infants, but the origin of the bacteria found in HM still remains unclear [17]. Additionally, the microbiota in HM is quite variable among women and has been shown to be influenced by several maternal and environmental factors [58]; however, little research is available regarding how the HM microbiota differs among mothers utilizing different feeding modes, e.g., full or partial breastfeeding. Insight into the origin of milk bacteria, factors influencing its composition, and its contribution to the infant gut microbiota are necessary in order to further understand how gut microbial communities are shaped during infancy. This study investigated the microbial relationships between human milk (HM), breast skin (SK), infant saliva (ISa), maternal saliva (MSa), maternal stool (MSt), and infant stool (ISt) of mother–infant pairs at 6 wks postpartum and evaluated how these communities differ among EBF and MF dyads.

Our findings demonstrate that the composition of each sampled maternal and infant body site is distinct, with MSa and MSt demonstrating the greatest diversity. Among our participants, feeding mode had the greatest impact on ISa compared to other microbial communities. Infants consuming both HM and formula had a more diverse oral microbiota, with a higher abundance of *Veillonella* and lower abundances of *Streptococcus* and *Gemella*, which is consistent with previous results comparing EBF to formula-fed infants [37,38]. The differences between feeding groups could be attributed to less exposure to the HM microbiota or perhaps less exposure to antimicrobials known to be present in HM. Feeding mode had little effect on the bacterial composition of SK or HM, which may be partially attributed to the small sample size and large variability in these communities. However, *Streptococcus* abundance did tend to be higher in the HM of EBF mothers, which likely reflects the higher abundance of this bacterium in the ISa of EBF infants. Recent findings show a greater abundance of *Acinetobacter* and reduced abundance of *Pseudomonas* in the HM of partially breastfeeding mothers compared to EBF mothers at 3 months postpartum [39]; however, we did not conduct differential abundance analysis of these genera due to low prevalence in our samples. The overall microbial structure of HM, SK, and ISa differed in our study; however, these three communities harbored several common bacterial genera, such as *Staphylococcus*, *Streptococcus*, *Rothia, Gemella*, and *Veillonella*, likely exhibiting the interaction of these microbial communities during feeding at the breast. Additionally, we found no effect of feeding mode on the abundance of genera detected in ISt. The gut microbiota composition of MF infants is known to be variable, with some infants clustering closer to EBF infants and some closer to formula-fed [7,32]. Proportions of feedings per day from HM varied greatly among MF infants in our study (10–90%), likely influencing our ability to detect compositional differences due to feeding mode.

Our data using SourceTracker2 and FEAST demonstrated that each sampled site was predicted to contribute at least a small percentage of ASVs to HM, collectively accounting for over half of the ASVs in milk. Among microbial sources sampled in our study, SK was predicted to contribute the largest fraction of ASVs to HM followed by ISa. The lack of compositional differences in HM between the feeding groups may be partially explained by the large contribution of SK, the composition of which was not different between EBF and MF mothers. Utilizing SourceTracker2, Williams et al. predicted a much larger percentage of bacteria in HM to come from ISa than in our study [31]; however, SK was not included in their analysis, which may account for this inconsistency. A substantial proportion of ASVs in HM was predicted to come from unknown sources, which could be other maternal body sites, individuals in the home, breast pumps, or other environmental sources. Given that they were predicted to contribute over 25% of the bacterial community in HM in our study, these unknown sources may be, in part, responsible for the great variation observed in HM microbiota composition among mothers. While FEAST was able to predict microbial sources for a greater fraction of HM bacteria, the pattern was comparable between the two methods. Quite surprisingly, we were unable to predict sources for almost 99% of the bacteria present in ISt with SourceTracker2, consistent with recent findings [31]. However, the percent contribution was greatly increased with FEAST analysis, suggesting that this may be a superior method for microbial source prediction for this community. In particular, FEAST estimated that a sizeable fraction of the ISt bacterial community arose from MSt followed by HM, though the majority of the ASVs in ISt were still predicted to come from unsampled sources. Bacteria shared between both HM [33] and MSt [23] and ISt are known to decrease considerably over the first few months postpartum. Retention of maternal strains within the infant gut may be determined by the fitness, not quantity, of the microorganisms delivered to the infant [23]. Hypothetically, the gut microbiota of infants in our study may be, in part, comprised of maternal strains vertically transmitted during delivery or the early postpartum period that are no longer present in the maternal microbiome at 6 wks postpartum. The additional members of the microbiota in ISt are likely a result of transient colonization from environmental sources as oxygen levels in the gastrointestinal tract decrease [23] or transferred from other individuals in the home, whom we did not sample.

Lastly, we assessed bacterial networks between HM and other microbial sites of mothers and infants and found several relationships among sampled communities. Though SourceTracker2 and FEAST analyses estimated that HM only contributed a small fraction of ASVs to ISt, the greatest number of co-occurrence patterns were found between bacteria in these two sites. These relationships, however, were not shown between the same bacterial genera, supporting the concept proposed by Williams and colleagues that HM microbiota may affect the proximal gastrointestinal tract, thereby indirectly affecting the microbiota of the distal colon and feces [31]. Relationships between the same genera were only apparent in HM and SK and ISa, likely due to the proximity or other environmental characteristics of these microbial sites. Mechanisms underlying the associations detected between HM and MSt genera are unclear. All of the MSt genera shown to co-occur with HM bacteria are members of the Firmicutes phylum and, other than *Dialister*, were present in MSt at low mean relative abundance (<1%). These stool genera that co-occurred with others in HM could be biomarkers of maternal diet or health status or may produce metabolites that are delivered to the mammary gland, all of which may influence the HM microbiota. Co-occurrence relationships between MSt and HM communities may highlight the potential for modulation of the maternal gut microbiota as a method to indirectly alter the bacterial composition of HM. While MSt was estimated to contribute the smallest fraction of ASVs to HM, these findings do not refute the notion of enteromammary trafficking of bacteria; however, they may prompt investigators to explore novel strategies to modulate the HM microbiota. Overall, whether the identified co-occurrence relationships are indicative of proximity, mutualism, or similar responses to environmental stimuli or are biomarkers of host–microbe interactions remains to be determined.

This study is not without limitations. The sample size was small, and the proportion of feedings per day from HM by MF infants was quite variable, both of which likely impacted our ability to detect differences in bacterial composition between feeding groups. Though we originally intended to include exclusively formula-feeding infants as a reference group, we were unable to recruit a sufficient number of dyads. In the future, incorporating formula-fed infants into similar studies will help to elucidate the impact of the HM microbiota on infant gut microbiota development and further highlight the impact of breastfeeding on other microbial communities of mothers and infants. Another limitation is our lack of DNA extraction controls. While negative dilution and sequencing controls showed negligible read counts (<100), we are not able to identify potential contaminants that may have been introduced during extraction, which is a concern given the low biomass of some samples included in this study.

In summary, our results highlight several associations between microbial communities of breastfeeding dyads. Findings demonstrate that multiple maternal and infant microbial communities collectively contribute to the HM microbiota, with SK being a primary source. We identified unique ecological relationships between HM and other microbial sites of breastfeeding dyads. In addition to vertical or bi-directional transfer, future studies should investigate interactions among microbial habitats of mothers and infants. Lastly, even with a small sample size, we demonstrated robust differences in the overall microbial structure and taxonomy of ISa between EBF and MF infants. While there is some emerging work on the infant oral microbiome, it is still an understudied microbial community. Sample collection from the infant oral cavity is relatively non-invasive and may provide a window into how bacteria or other components infants are exposed to via HM influence the mucosal immune system. Further understanding of mechanisms driving microbial community assembly will help to uncover potential targets for microbial modulation and guide the development of microbiota-tailored interventions to promote the development of a healthy infant microbiome, thereby influencing long-term health and risk for disease.

## Figures and Tables

**Table 1:** Demographic characteristics.

Characteristic	EBF (*n* = 20)	MF (*n* = 13)
Maternal age (years)	32.1 (5.3)	33.0 (3.8)
Maternal BMI (kg/m^2^)		
Pre-pregnancy	22.4 (3.0) ^a^	27.4 (7.9) ^b^
6-weeks postpartum	24.5 (4.2)	26.1 (14.7)
Delivery mode		
Vaginal delivery	18 (90) ^a^	6 (46) ^b^
Planned C-section	1 (5)	2 (15)
Emergency C-section	1 (5)	5 (38)
Infant gender		
Male	9 (45)	9 (69)
Female	11 (55)	4 (31)
Percent feedings from HM	100	67.0 (35.0)
Percent feedings from formula	0	33.0 (35.0)
Consumed prenatal probiotics	3 (15)	0
Regular pumping at 6-weeks		
Yes	9 (45)	7 (58)
No	9 (45)	5 (42)
Unknown	2 (10)	1 (3)

Values are expressed as a number (%) or median (IQR). Continuous data were analyzed by Kruskal–Wallis and categorical data were analyzed by Fisher’s exact test. ^a,b^ Values within the same row not sharing a common superscript differ, *p* < 0.05. EBF, exclusively breastfed; MF, mixed-fed; HM, human milk; C-section, cesarean section.

**Table 2:** Alpha diversity by sample type and feeding group.

	Shannon Index	Observed Features	Faith Phylogenetic Diversity
Human milk			
EBF (*n* = 20)	2.3 ± 0.2	22.9 ± 1.9	18.2 ± 1.5
MF (*n* = 13)	2.6 ± 0.3	29.2 ± 4.0	14.3 ± 1.8
Breast skin			
EBF (*n* = 20)	3.0 ± 0.1 ^a^	41.9 ± 3.9	10.2 ± 0.4
MF (*n* = 13)	3.5 ± 0.3 ^b^	52.0 ± 6.7	10.1 ± 0.5
Infant saliva			
EBF (*n* = 20)	2.1 ± 0.2 ^a^	25.4 ± 1.3 ^a^	13.8 ± 1.0
MF (*n* = 13)	3.1 ± 0.2 ^b^	36.7 ± 3.8 ^b^	13.6 ± 1.3
Maternal saliva			
EBF (*n* = 18)	5.3 ± 0.1	152.8 ± 5.6	14.7 ± 0.4
MF (*n* = 13)	5.3 ± 0.1	160.6 ± 9.5	15.5 ± 1.0
Infant stool			
EBF (*n* = 20)	2.6 ± 0.2	22.8 ± 1.5	6.9 ± 0.1
MF (*n* = 12)	2.5 ± 0.2	24.7 ± 1.8	7.3 ± 0.2
Maternal stool			
EBF (*n* = 18)	5.2 ± 0.1	101.2 ± 4.9	12.4 ± 0.3
MF (*n* = 13)	4.9 ± 0.2	94.2 ± 6.9	11.9 ± 0.5

Data are presented as mean ± SEM. Normally distributed data were analyzed with the MIXED procedure. Non-normally distributed data were analyzed with the GLIMMIX procedure. Models run for human milk, breast skin, infant saliva, and infant stool included delivery mode as a covariate. Models run for maternal saliva and maternal stool included BMI at 6 wks postpartum as a covariate. ^a,b^ Values within a body site and diversity index not sharing a common superscript differ at *p* ≤ 0.05. EBF, exclusively breastfed; MF, mixed-fed.

**Table 3:** Relative abundance (%) of the top 10 most abundant bacterial genera present on each maternal and infant body site by feeding mode.

Genus	Human Milk		Breast Skin		Infant Saliva		Infant Stool		Maternal Saliva		Maternal Stool	
	EBF	MF	EBF	MF	EBF	MF	EBF	MF	EBF	MF	EBF	MF
*Staphylococcus*	45.4 ± 6.8	48.8 ± 9.9	32.7 ± 5.3	37.5 ± 8.2	0.4 ± 0.2	1.0 ± 0.8						
***Streptococcus***	33.4 ± 5.9 ^†^	19.0 ± 5.9	41.7 ± 4.0	31.4 ± 5.1	67.4 ± 3.5 *	53.5 ± 4.5	2.1 ± 0.6	2.3 ± 1.1	17.3 ± 1.5	19.3 ± 2.1		
*Corynebacterium 1*	4.1 ± 1.8	2.9 ± 1.2	7.1 ± 1.6	5.4 ± 1.2								
*Rothia*	3.5 ± 1.6	1.3 ± 0.4	2.0 ± 0.5	2.6 ± 1.2	3.6 ± 1.3	9.6 ± 3.1			2.9 ± 0.6	3.2 ± 0.7		
***Gemella***	1.9 ± 1.0	0.6 ± 0.4	3.0 ± 0.9	1.5 ± 0.6	15.1 ± 2.3 *	7.9 ± 2.0						
***Veillonella***	1.1 ± 0.7	1.8 ± 1.4	2.0 ± 0.5	3.1 ± 0.8	3.9 ± 1.2 *	10.7 ± 2.1	4.0 ± 1.9	4.7 ± 3.7	6.3 ± 0.7	6.0 ± 1.1		
*Cutibacterium*	1.3 ± 0.7	0.7 ± 0.4										
*Bifidobacterium*	0.2 ± 0.2	0.6 ± 0.6					17.1 ± 4.7	31.2 ± 6.7			3.1 ± 1.3	2.1 ± 0.7
*Actinomyces*	0.2 ± 0.1	0.3 ± 0.2	1.0 ± 0.4	1.1 ± 0.5								
*Streptomyces*	0.3 ± 0.1	0.1 ± 0.1										
*Lactobacillus*			2.0 ± 1.9	0.4 ± 0.2								
*Prevotella 7*			0.4 ± 0.4	1.9 ± 1.3	0.4 ± 0.3	2.5 ± 1.8			17.1 ± 1.7	13.5 ± 1.4		
*Haemophilus*			0.6 ± 0.4	1.5 ± 1.1	5.2 ± 2.4	2.9 ± 2.6			10.0 ± 1.3	7.7 ± 1.2		
*Atopobium*					1.0 ± 0.8	1.3 ± 1.0						
*Neisseria*					0.0 ± 0.0	2.4 ± 2.4			8.7 ± 1.9	11.7 ± 2.3		
*Porphyromonas*					0.5 ± 0.5	0.8 ± 0.5			3.3 ± 0.9	3.4 ± 0.8		
*Escherichia-Shigella*							16.0 ± 5.0	17.1 ± 6.3			2.1 ± 2.1	4.5 ± 3.1
***Bacteroides***							17.0 ± 5.1	7.0 ± 4.9			17.0 ± 2.2 ^†^	18.7 ± 3.9
*Klebsiella*							9.4 ± 3.9	6.8 ± 4.0				
*Clostridium sensu stricto 1*							7.2 ± 3.1	0.8 ± 0.5				
*Enterobacter*							6.2 ± 3.6	2.1 ± 1.5				
*Enterococcus*							2.0 ± 0.9	5.3 ± 3.7				
*Ruminococcus gnavus group*							0.6 ± 0.4	4.0 ± 2.6				
***Fusobacterium***									6.4 ± 1.2 *	10.0 ± 1.2		
*Alloprevotella*									5.0 ± 0.5	4.1 ± 0.8		
*Leptotrichia*									2.7 ± 0.5	3.1 ± 1.4		
*Faecalibacterium*											12.2 ± 2.1	9.9 ± 2.4
*Blautia*											7.2 ± 1.1	9.8 ± 2.3
*Akkermansia*											4.8 ± 1.6	5.3 ± 2.2
*Subdoligranulum*											3.5 ± 1.2	3.4 ± 1.0
*Eubacterium hallii group*											3.6 ± 0.6	3.1 ± 0.9
*Ruminococcus 2*											3.5 ± 1.1	2.7 ± 0.9
*Dialister*											1.1 ± 0.6	4.4 ± 2.6

N = 33 mothers and infants (EBF = 20, MF = 13); infant stool, MF = 12. Data are expressed mean ± SEM and were analyzed using the GLIMMMIX procedure. For human milk, breast skin, infant saliva, and infant stool analyses, delivery mode was included as a covariate. For maternal saliva and stool, maternal BMI at 6 wks postpartum was included as a covariate. Values within the same row and body site are significantly different at * *p* ≤ 0.05; **^†^** *p* ≤ 0.1. Bolded genus name signifies statistical difference or trend between groups on at least one body site. EBF, exclusively breastfed; MF, mixed-fed.

## Data Availability

All data analyzed in this study are available upon request.

## Supplementary Materials

The following supporting information can be downloaded at: https://www.mdpi.com/article/10.3390/microorganisms10061155/s1, Table S1. Beta diversity measures between feeding groups by sample type. Tables S2–S7. Relative abundance of most abundant bacterial genera present on maternal and infant body sites. Table S8. Proportion of ASVs (% of total) that different bacterial communities are predicted to contribute to the bacterial composition of human milk and infant stool.
