# Supplementary material for: Microbial Interrelationships across Sites of Breastfeeding Mothers and Infants at 6 Weeks Postpartum
Source: Microorganisms. 2022 Jun 2;10(6):1155. doi: 10.3390/microorganisms10061155 (PMC9230604; doi:10.3390/microorganisms10061155)
Supplement: Supplementary file 1 [file microorganisms-10-01155-s001.zip › microorganisms-1730087-supplementary.pdf]

Table S1. Beta diversity measures between feeding groups by sample type

	Unweighted UniFrac	Weighted UniFrac	Bray-Curtis Dissimilarity
		p-value	
Human milk	0.05	0.05	0.57
Breast skin	0.59	0.21	0.17
Infant saliva	0.72	0.004	0.001
Maternal saliva	0.54	0.06	0.27
Infant stool	0.06	0.15	0.37
Maternal stool	0.37	0.93	0.36

N=33 dyads (EBF=20, MF=13). Data were analyzed by PERMANOVA.

Table S2. Relative abundance (%) of bacterial genera in human milk samples by feeding mode

Genus	EBF	MF
Staphylococcus	45.4 ± 6.8	48.8 ± 9.9
Streptococcus†	33.4 ± 5.9	19.0 ± 5.9
Corynebacterium 1	4.1 ± 1.8	2.9 ± 1.2
Rothia	3.5 ± 1.6	1.3 ± 0.4
Gemella	1.9 ± 1.0	0.6 ± 0.4
Veillonella	1.1 ± 0.7	1.8 ± 1.4
Cutibacterium	1.3 ± 0.7	0.7 ± 0.4
Bifidobacterium	0.2 ± 0.2	0.6 ± 0.6
Actinomyces	0.2 ± 0.1	0.3 ± 0.2
Streptomyces	0.3 ± 0.1	0.1 ± 0.1

N=33 mothers (EBF=20, MF=13). Only genera present in ≥ 20% of samples and at ≥ 0.1% abundance are included in the table. Data are expressed mean ± SEM and were analyzed using the GLIMMMIX procedure including delivery mode as a covariate. Values within the same row are significantly different at *p≤ 0.5; †p≤ 0.1. EBF, exclusively breastfeeding; MF, mixed-feeding.

Table S3. Relative abundance (%) of 20 most abundant bacterial genera on breast skin by feeding mode

Genus	EBF	MF
Streptococcus	41.7 ± 4.0	31.4 ± 5.1
Staphylococcus	32.7 ± 5.3	37.5 ± 8.2
Corynebacterium 1	7.1 ± 1.6	5.4 ± 1.2
Gemella	3.0 ± 0.9	1.5 ± 0.6
Veillonella	2.0 ± 0.5	3.1 ± 0.8
Rothia	2.0 ± 0.5	2.6 ± 1.2
Lactobacillus	2.0 ± 1.9	0.4 ± 0.2
Prevotella 7	0.4 ± 0.4	1.9 ± 1.3
Actinomyces	1.0 ± 0.4	1.1 ± 0.5
Haemophilus	0.6 ± 0.4	1.5 ± 1.1
Atopobium	0.5 ± 0.2	1.4 ± 0.7
Acinetobacter	0.6 ± 0.2	1.0 ± 0.4
Micrococcus	0.6 ± 0.3	0.7 ± 0.3
Cutibacterium	0.5 ± 0.1	0.9 ± 0.4
Anaerococcus†	0.2 ± 0.1	0.9 ± 0.3
Bacteroides	0.3 ± 0.1	0.7 ± 0.3
Bifidobacterium	0.2 ± 0.1	0.8 ± 0.6
Enhydrobacter	0.4 ± 0.2	0.3 ± 0.1
Neisseria	0.1 ± 0.0	0.3 ± 0.2
Lawsonella	0.2 ± 0.1	0.3 ± 0.2

N=33 mothers (EBF=20, MF=13). Only genera present in $\geq$ 20% of samples and at $\geq$ 0.1% abundance are included in the table. Data are expressed mean ± SEM and were analyzed using the GLIMMMIX procedure including delivery mode as a covariate. Values within the same row are significantly different at *$p \leq 0.5$; †$p \leq 0.1$. EBF, exclusively breastfeeding; MF, mixed-feeding.

Table S4. Relative abundance (%) of bacterial genera in infant saliva by feeding mode

Genus	EBF	MF
*Streptococcus**	67.4 ± 3.5	53.5 ± 4.5
*Gemella**	15.1 ± 2.3	7.9 ± 2.0
*Veillonella**	3.9 ± 1.2	10.7 ± 2.1
Rothia	3.6 ± 1.3	9.6 ± 3.1
Haemophilus	5.2 ± 2.4	2.9 ± 2.6
Prevotella 7	0.4 ± 0.3	2.5 ± 1.8
Atopobium	1.0 ± 0.8	1.3 ± 1.0
Neisseria	0.0 ± 0.0	2.4 ± 2.4
Porphyromonas	0.5 ± 0.5	0.8 ± 0.5
Staphylococcus	0.4 ± 0.2	1.0 ± 0.8
Bergeyella	0.5 ± 0.2	0.8 ± 0.3
Actinomyces	0.4 ± 0.2	0.6 ± 0.4

N=33 infants (EBF=20, MF=13). Only genera present in $\geq$ 20% of samples and at $\geq$ 0.1% abundance are included in the table. Data are expressed mean ± SEM and were analyzed using the GLIMMMIX procedure including delivery mode as a covariate. Values within the same row are significantly different at *p$\leq$ 0.5; †p$\leq$ 0.1. EBF, exclusively breastfeeding; MF, mixed-feeding.

Table S5. Relative abundance (%) of bacterial genera in infant stool by feeding mode

Genus	EBF	MF
Bifidobacterium	17.1 ± 4.7	31.2 ± 6.7
Escherichia-Shigella	16.0 ± 5.0	17.1 ± 6.3
Bacteroides	17.0 ± 5.1	7.0 ± 4.9
Klebsiella	9.4 ± 3.9	6.8 ± 4.0
Clostridium sensu stricto 1	7.2 ± 3.1	0.8 ± 0.5
Enterobacter	6.2 ± 3.6	2.1 ± 1.5
Veillonella	4.0 ± 1.9	4.7 ± 3.7
Enterococcus	2.0 ± 0.9	5.3 ± 3.7
Streptococcus	2.1 ± 0.6	2.3 ± 1.1
Ruminococcus gnavus group	0.6 ± 0.4	4.0 ± 2.6
Haemophilus	2.0 ± 1.2	0.6 ± 0.6
Staphylococcus	0.7 ± 0.3	0.2 ± 0.1
Lactobacillus	0.5 ± 0.3	0.2 ± 0.1
Eggerthella	0.0 ± 0.0	0.9 ± 0.6

N=32 infants (EBF=20, MF=12). Only genera present in $\geq$ 20% of samples and at $\geq$ 0.1% abundance are included in the table. Data are expressed mean ± SEM and were analyzed using the GLIMMMIX procedure including delivery mode as a covariate. Values within the same row are significantly different at *p$\leq$ 0.5; †p$\leq$ 0.1. EBF, exclusively breastfeeding; MF, mixed-feeding.

Table S6. Relative abundance (%) of 20 most abundant bacterial genera in maternal saliva by feeding mode

Genus	EBF	MF
Streptococcus	17.3 ± 1.5	19.3 ± 2.1
Prevotella 7	17.1 ± 1.7	13.5 ± 1.4
Neisseria	8.7 ± 1.9	11.7 ± 2.3
Haemophilus	10.0 ± 1.3	7.7 ± 1.2
*Fusobacterium**	6.4 ± 1.2	10.0 ± 1.2
Veillonella	6.3 ± 0.7	6.0 ± 1.1
Alloprevotella	5.0 ± 0.5	4.1 ± 0.8
Porphyromonas	3.3 ± 0.9	3.4 ± 0.8
Rothia	2.9 ± 0.6	3.2 ± 0.7
Leptotrichia	2.7 ± 0.5	3.1 ± 1.4
Actinomyces	2.5 ± 0.4	2.2 ± 0.4
Prevotella	2.6 ± 0.4	1.6 ± 0.2
Campylobacter	1.5 ± 0.3	2.4 ± 0.6
*Saccharimonadaceae uncultured**	2.0 ± 0.3	1.0 ± 0.3
Atopobium	1.6 ± 0.4	1.3 ± 0.3
Gemella	0.9 ± 0.1	1.4 ± 0.4
*Granulicatella**	1.1 ± 0.1	0.8 ± 0.1
Absconditabacteriales uncultured	1.0 ± 0.5	0.5 ± 0.2
Prevotella 6†	0.9 ± 0.1	0.5 ± 0.1
Lachnoanaerobaculum	0.5 ± 0.1	0.4 ± 0.0

N=33 mothers (EBF=20, MF=13). Only genera present in $\geq$ 20% of samples and at $\geq$ 0.1% abundance are included in the table. Data are expressed mean ± SEM and were analyzed using the GLIMMMIX procedure including BMI at 6 wk postpartum as a covariate. Values within the same row are significantly different at *****$p \leq 0.5$; $^{\dagger}p \leq 0.1$. EBF, exclusively breastfeeding; MF, mixed-feeding.

Table S7. Relative abundance (%) of 20 most abundant bacterial genera present in maternal stool by feeding mode

Genus	EBF	MF
Bacteroides†	17.0 ± 2.2	18.7 ± 3.9
Faecalibacterium	12.2 ± 2.1	9.9 ± 2.4
Blautia	7.2 ± 1.1	9.8 ± 2.3
Akkermansia	4.8 ± 1.6	5.3 ± 2.2
Subdoligranulum	3.5 ± 1.2	3.4 ± 1.0
Eubacterium hallii group	3.6 ± 0.6	3.1 ± 0.9
Ruminococcus 2	3.5 ± 1.1	2.7 ± 0.9
Escherichia-Shigella	2.1 ± 2.1	4.5 ± 3.1
Bifidobacterium	3.1 ± 1.3	2.1 ± 0.7
Dialister	1.1 ± 0.6	4.4 ± 2.6
Alistipes	2.2 ± 0.4	2.1 ± 0.7
Agathobacter	2.0 ± 0.5	1.7 ± 0.7
Anaerostipes	2.2 ± 0.3	1.4 ± 0.4
Collinsella	1.4 ± 0.6	1.8 ± 0.6
Ruminiclostridium 5†	0.8 ± 0.2	2.6 ± 1.0
Ruminococcus 1	2.1 ± 0.4	0.6 ± 0.3
Ruminococcus torques group	1.4 ± 0.2	1.5 ± 0.3
Lachnospiraceae	1.8 ± 0.4	1.0 ± 0.2
Erysipelotrichaceae UCG-003	1.8 ± 0.5	0.8 ± 0.3
Roseburia	1.9 ± 0.3	0.6 ± 0.2

N=33 mothers (EBF=20, MF=13). Only genera present in ≥ 20% of samples and at ≥ 0.1% abundance are included in the table. Data are expressed mean ± SEM and were analyzed using the GLIMMMIX procedure including BMI at 6 wk postpartum as a covariate. Values within the same row are significantly different at *p≤ 0.5; †p≤ 0.1. EBF, exclusively breastfeeding; MF, mixed-feeding.

Table S8. Proportion of ASVs (% of total) that different bacterial communities are predicted to contribute to bacterial composition of human milk and infant stool

Source	SourceTracker2	FEAST- Individual	FEAST- Group
**human milk**			
Breast skin	50.0 ± 6.1	57.8 ± 4.7	59.5 ± 5.6
Infant saliva	9.0 ± 3.3	11.1 ± 3.0	16.2 ± 3.7
Maternal stool	0.04 ± 0.0	0.03 ± 0.02	0.5 ± 0.3
Maternal saliva	0.4 ± 0.1	1.0 ± 0.2	0.3 ± 0.1
Unknown	40.6 ± 5.9	30.0 ± 4.6	23.4 ± 5.0
**infant stool**			
Human milk	0.4 ± 0.1	6.3 ± 2.7	3.7 ± 1.2
Breast skin	0.3 ± 0.1	5.8 ± 3.1	1.5 ± 0.4
Infant saliva	0.3 ± 0.1	3.8 ± 1.5	2.1 ± 0.7
Maternal stool	0.1 ± 0.0	12.7 ± 4.2	28.3 ± 4.4
Maternal saliva	0.2 ± 0.0	1.5 ± 1.0	2.0 ± 0.8
Unknown	98.8 ± 0.2	69.9 ± 5.4	62.5 ± 4.1

Human milk, n=33; infant stool, n=32. Data are expressed mean ± SEM. ASV, amplicon sequence variant.
